# General anesthesia with remimazolam in a patient with clinically suspected malignant hyperthermia

**DOI:** 10.1186/s40981-021-00482-4

**Published:** 2021-10-18

**Authors:** Keita Uchiyama, Hiroshi Sunaga, Nobuyuki Katori, Shoichi Uezono

**Affiliations:** Department of Anesthesiology, The Jikei University School of Medicine, 3-25-8 Nishishimbashi, Minatoku, Tokyo, 105-8461 Japan

To the Editor,

Malignant hyperthermia (MH) is a life-threatening complication of general anesthesia. Avoidance of the trigger drugs is mandatory for known or suspected MH-susceptible patients, and total intravenous anesthesia (TIVA) should be chosen if general anesthesia is necessary [1]. Whereas an in vitro study suggested that a new ultra-short-acting benzodiazepine, remimazolam, might be safely used without causing MH [2], our literature search failed to identify any reports of remimazolam use for MH in clinical practice. We present a case of a patient with a medical history of suspected MH safely managed under general anesthesia with remimazolam. Informed consent was obtained from the patient for this publication.

The patient was a 26-year-old man (177 cm, 64 kg) who was scheduled to undergo endoscopic sinus surgery for allergic rhinitis and a septoplasty for a deflected nasal septum. He underwent open reduction and internal fixation for a right forearm fracture under general anesthesia at another hospital at the age of 11 years. He developed a high temperature, reddish-brown urine, and hyperkalemia during that surgery, and a clinical diagnosis of MH was made. The operation was finished quickly, and he was treated in the intensive care unit. Although a specialized test for definitive diagnosis of MH susceptibility was not performed, the high level of the creatine kinase (approximately 40,000 IU/L) and myoglobinuria (approximately 3000 ng/mL) after surgery supported a clinical diagnosis of MH. He did not undergo general anesthesia since that time and underwent removal of the internal fixation with several rounds of local anesthesia. His past medical history also included an allergic reaction to soy.

For the procedure, an anesthesia machine was flushed and prepared according to the manufacturer’s recommendations [3]. Anesthesia was induced with an infusion of remimazolam at a rate of 12 mg/kg/h along with rocuronium, fentanyl, and remifentanil, and the trachea was intubated. The patient was mechanically ventilated, and anesthesia was maintained with an infusion of remimazolam at 1.5 mg/kg/h along with rocuronium, fentanyl, and remifentanil. Body temperature remained at 36.3°C to 36.9°C, and there were no clinical signs of MH during anesthesia. The infusions of remimazolam and remifentanil were discontinued at the end of surgery. Sugammadex and flumazenil were administered, and the trachea was extubated. The surgery took 2 h and 13 min, and the anesthesia time was 2 h and 59 min. Monitoring and observation were continued in the postanesthesia care unit, and the patient was then transferred to the general ward. No signs of MH were noted until discharge from the hospital on the second postoperative day.

Propofol can be administered without any concerns for patients with MH susceptibility and patients allergic to soy [4, 5]. That said, remimazolam should not be inferior to propofol with respect to efficacy as a sedative agent for general anesthesia and might have fewer hemodynamic side effects [6]. We considered that remimazolam might be beneficial for TIVA owing to reversibility with flumazenil or avoidance of propofol infusion syndrome. This case suggests that patients with clinically suspected MH can be safely managed under general anesthesia with remimazolam.

## Data Availability

Not applicable.

## Abbreviations

MH: Malignant hyperthermia
TIVA: Total intravenous anesthesia
